# Detecting taxonomic and phylogenetic signals in equid cheek teeth: towards new palaeontological and archaeological proxies

**DOI:** 10.1098/rsos.160997

**Published:** 2017-04-05

**Authors:** T. Cucchi, A. Mohaseb, S. Peigné, K. Debue, L. Orlando, M. Mashkour

**Affiliations:** 1 CNRS, Muséum national d'Histoire naturelle, Sorbonne Universités, UMR 7209, Archéozoologie, Archéobotanique: Sociétés, Pratiques et Environnements, 75005 Paris, France; 2 Department of Archaeology, University of Aberdeen, St Mary's, Aberdeen, UK; 3 UMR 7207 Centre de recherche sur la paléobiodiversité et les paléoenvironnements (CR2P), MNHN/CNRS/Univ. Paris 06, CP/38, 8 rue Buffon, 75005 Paris, France; 4 Centre for GeoGenetics, Natural History Museum of Denmark, University of Copenhagen, Øster Voldgade 5-7, 1350 K Copenhagen, Denmark; 5 Laboratoire d'Anthropobiologie Moléculaire et d'Imagerie de Synthèse, Université de Toulouse, University Paul Sabatier, CNRS UMR 5288, 31000 Toulouse, France

**Keywords:** *Equus*, phylogenetic signal, shape, tooth geometric morphometrics, equid evolutionary history, fossil record

## Abstract

The Plio–Pleistocene evolution of *Equus* and the subsequent domestication of horses and donkeys remains poorly understood, due to the lack of phenotypic markers capable of tracing this evolutionary process in the palaeontological/archaeological record. Using images from 345 specimens, encompassing 15 extant taxa of equids, we quantified the occlusal enamel folding pattern in four mandibular cheek teeth with a single geometric morphometric protocol. We initially investigated the protocol accuracy by assigning each tooth to its correct anatomical position and taxonomic group. We then contrasted the phylogenetic signal present in each tooth shape with an exome-wide phylogeny from 10 extant equine species. We estimated the strength of the phylogenetic signal using a Brownian motion model of evolution with multivariate *K* statistic, and mapped the dental shape along the molecular phylogeny using an approach based on squared-change parsimony. We found clear evidence for the relevance of dental phenotypes to accurately discriminate all modern members of the genus *Equus* and capture their phylogenetic relationships. These results are valuable for both palaeontologists and zooarchaeologists exploring the spatial and temporal dynamics of the evolutionary history of the horse family, up to the latest domestication trajectories of horses and donkeys.

## 1. Introduction

Extant living species of equids are assigned to the genus *Equus* and are spread across large and diverse environments in Africa and Eurasia. The *Equus* genus is made up of two lineages: the caballine and non-caballine horses, and three phylogenetic clades. The first clade encompasses the caballine lineage that includes domesticated horses (*Equus ferus caballus*) and the endangered Przewalski's horse (*Equus ferus przewalskii*), which is the only remaining population of truly wild horses on the planet [1]. Zebras and wild asses form the two other remaining clades and belong to the non-caballine lineage. Zebras include three species plus a number of subspecies/morphotypes. Wild asses include the African wild ass *(Equus africanus)* and its domestic form the donkey (*Equus africanus asinus*), the Asian wild asses, *Equus kiang* and *E. hemionus*, the latter comprising several recognized subspecies [2–6]. This extant diversity is also made up of hybrids, as equine species can be crossbred with each other but remain sterile (although with some rare exceptions [7]). The most common hybrids are mules, a cross between a male donkey and a female horse, and to a lesser extent hinnies, the reciprocal mating.

The relatively limited biodiversity present in contemporary equine species appears in striking contrast with the past diversity of the Equidae family, in which several dozen genera of species have been identified from a fossil record spanning the last 55 Myr [8]. Most of this diversity emerged during an adaptive diversification stage which took place in the second half of the Miocene, following the increased range of new biomes and the transition from browsing to grazing [9]. The *Equus* genus, which represents the only genus of living equids, emerged approximately 4.0–4.5 Myr ago in Northern America, and then spread into the Old World 2.1–3.4 Myr ago [5,10] where the zebra and ass clades emerged. Caballine horses crossed the Bering Strait much later before expanding into the Old World; they became extinct in Northern America some 10 000 years ago [11].

Members of the *Equus* genus were abundant and diverse during the European Pleistocene [12], but almost became extinct at the beginning of the Holocene due to a combination of several possible factors, including climate change and overhunting. However, the *Equus* species have persisted in small ranges until now; with two of these being domesticated around 6000 years ago—in the west side of the Eurasian Steppe for the horse [13] and in Northern Africa for the donkey [14]—although the domestication processes of both [14–19] remain poorly understood.

Although the evolutionary history of the equid family provides one classical textbook example in palaeontology, our knowledge of a number of key evolutionary stages, such as the Plio–Pleistocene evolution of *Equus*, especially in America [20,21], is incomplete. Understanding how equine species spread through space and time requires the development of methods enabling robust identification from material preserved in the fossil record [22,23]. However, such identifications remain challenging for palaeontologists [8,24] and archaeologists [25], mostly due to the fragmentary nature of the material, convergent evolution and environmentally driven morphological changes, all of which limit the recoverable phylogenetic signal.

So far, ancient DNA analyses represent the ultimate method to secure an accurate taxonomic identification of ancient equids [1,5,6,20]. However, this approach is restricted to areas and/or periods conducive to the preservation of ancient DNA [26]; this would usually—but not always—exclude warm environments such as the deserts of the Middle East, Western Asia and Africa [27,28], though there are some exceptions [29,30]. Clearly, an integrative approach combining molecular and morphological proxies [31,32] would largely improve our understanding of the macro and micro evolutionary dynamics underlying equine speciation and domestication.

Teeth provide standard material to palaeontologists [33] and zooarchaeologists [34] for the reconstruction of past evolutionary changes. Teeth are well preserved in the fossil record and present relatively limited ecophenotypic plasticity, due to their genetically controlled development and absence of tissue remodelling once mineralized [35,36]. Crown morphology and the enamel pattern of mandibular cheek tooth occlusal surfaces are among the most popular morphological markers allowing palaeontologists and archaeologists to identify the taxonomy of Plio–Pleistocene equines [2,10,15,37,38], and reconstruct phylogenies and population histories [9,39]. The characters based on enamel patterns have long since been considered inappropriate to distinguish Pleistocene and Holocene horses [40], given that both age and wearing represent limiting factors for evolutionary [17] or taxonomic [25] reconstructions. However, with recent advances in geometric morphometrics (GMM) in the field of biometry [41–44], it is now possible to capture and graphically visualize the complexity of tooth morphology. When applied to the cheek teeth of modern horses, GMM has demonstrated statistically that age and wearing have no significant effects on the overall occlusal enamel shape pattern when excluding juvenile and senile specimens [45]. In addition, the basis of the shape of this occlusal enamel pattern was found to support a reliable assignment of the various source populations [45], which pleads for further investigations aimed at reconstructing the history of horse breeding.

Here, we assess the relevance of occlusal enamel patterns in the lower cheek teeth of extant Old World equids, collected to provide reliable interspecific taxonomic and phylogenetic signals. A phylogenetic signal can be observed when related species show close phenotypic relationships [46–48]. However, to test this signal we have chosen to map the underlying morphospace onto the whole-exome based phylogeny presented by Jónsson and co-workers [5], following previous approaches for the projection of a phylogenetic tree into geometric morphometric space [49,50], and estimate the strength of the phylogenetic signal in the morphometric dataset using multivariate *K* statistic [51]. Our first objective was to propose a single GMM protocol to capture the form of the occlusal enamel patterns of the four mandibular cheek teeth (P/3, P/4, M/1, M/2) used in species identification of equid remains [15,37]. Key to our GMM protocol was to perform a reliable assessment of the anatomical tooth position in order to enable future applications to the vast collections of isolated teeth available from the fossil record and museum collections. Our second objective was to find which mandibular cheek tooth captured the strongest interspecific differences and phylogenetic signal. Using dental phenotypes as reliable systematic and phylogenetic markers in equids would open up an incredibly large range of future investigations. Firstly, of the diversity of Plio–Pleistocene horses and, secondly, of the Holocene diversity of southwest Asian equids; where all three extant clades plus their hybrids and extinct forms could have occurred [29,52,53], and from which the wild ancestors of domesticated horses and donkeys emerged.

## 2. Material and methods

### 2.1. Material

**Figure 1.**
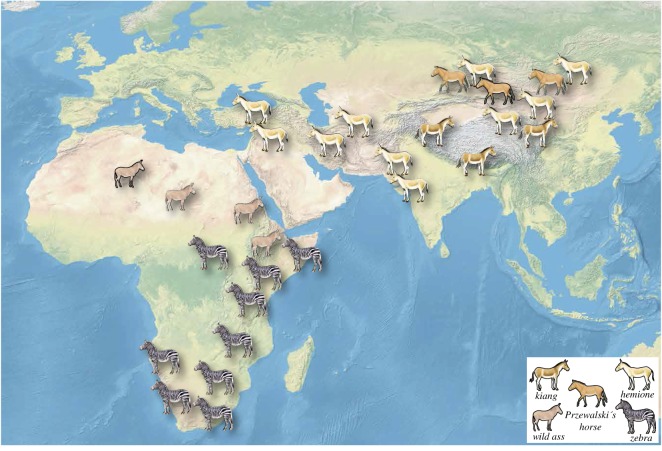
Distribution of extant clades of equids in Eurasia and Africa.

We studied a total of 345 specimens from 15 equine taxa covering the current distribution of all living members of the *Equus* genus (figure 1), including two taxa of horses, two taxa of African asses, six taxa of Asian asses, four taxa of zebras and one group of ass/horse hybrids (mules) (table 1; electronic supplementary material, S1). For each individual specimen, the occlusal view of the entire mandibular tooth row was photographed (electronic supplementary material, S1, details can be found also on www.vera-eisenmann.com). The occlusal surface of the mandibular cheek teeth were photographed using several film cameras (i.e. Hasselblad, Canon and Nikon), ensuring that the occlusal surface was parallel to the camera focal place. All the argentic photographs were scanned with a 300 dpi resolution, using a Toshiba e-studio 2330c scanner. Some of the specimens photographed within this project, were from the comparative anatomy collection of the National Museum of Natural History of Paris, using a Canon EOS 70D with Canon macro lens EF-S 60 mm.

**Table 1.** Equid taxa studied with their abbreviation code and their sample size per each mandibular cheek tooth.

taxa	vernacular names	code	P/3	P/4	M/1	M/2
*E. ferus caballus*	domestic horse	CBL	15	15	15	15
*E. f. przewalskii*	Przewalski's horse	PRZ	9	8	8	8
*E. africanus somaliensis*	Somali wild ass	AFR	7	7	7	7
*E. a. asinus*	domestic donkey	ASN	11	11	10	10
*E. kiang*	Tibetan kiang	KNG	12	13	13	13
*E. hemionus hemionus*	Mongolian wild ass	MNG	13	10	13	13
*E. h. onager*	onager	ONG	7	8	7	7
*E. h. khur*	Indian wild ass	KHR	7	8	8	6
*E. h. kulan*	kulan	KLN	9	12	10	11
*E. h. hemippus*	Syrian wild ass	HMP	2	3	2	3
*E. grevyi*	Grévy's zebra	GRV	3	3	3	3
*E. zebra hartmannae*	Hartmanns' mountain zebra	ZBR	4	4	4	5
*E. quagga quagga*	Quagga plain zebra	QGA	3	3	3	3
*E. q. burchellii*	Burchell's zebra	BRC	8	8	8	8
hybrid	donkey × horse	HBR	4	4	4	4

### 2.2. Geometric morphometrics

**Figure 2.**
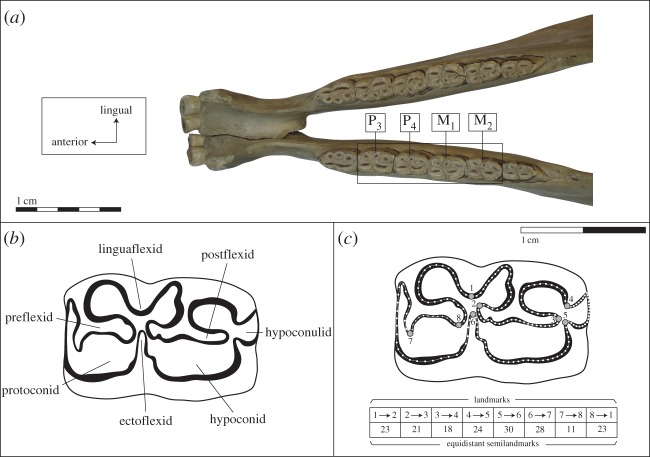
(*a*) Position of the four cheek teeth in the *Equus* mandible with (*b*) their anatomical terminology in occlusal view from [15] and (*c*) their common GMM protocol for the quantification of the enamel folding with the location of the 8 landmarks (dots with grey filling) and the number of semilandmarks (table) sampling the curves of the enamel folds between each landmarks.

From the picture of the mandibular tooth row from each specimen, the occlusal enamel patterns of P/3, P/4, M/1 and M/2 were recorded using the same novel two-dimensional protocol. This approach represents a significant increment compared with our previous study of the enamel pattern in equine mandibular teeth, which was based on a basic approach for capturing the complexity of the enamel folds with few landmarks on the maximum curvature of the enamel loops [45]. This study aimed to capture the entire complexity of the enamel folding by extracting as much biological information as possible (figure 2). More specifically, we used eight landmarks and eight curves, between each landmark, with a total of 178 equidistant semi-landmarks. Each curve was drawn in the centre of the enamel thickness (see details of the number of points per curves in figure 2). Only the left mandibles and teeth were used; all pictures of right mandibles were mirrored left.

Landmarks and semi-landmarks were digitized from images with a TPSdig2 v. 2.17 [54].

To be statistically comparable, enamel outlines must be standardized by a generalized Procrustes superimposition (GPS), which aligns their coordinates with a least squared approach in order to remove all information of position, scale and orientation from the initial points' configurations. In contrast with landmarks, semi-landmarks do not have an exact correspondence on the enamel curve. During the superimposition process, they are slid along the tangent of the curves using the bending energy algorithm [55] to minimize the difference between the shape configurations [56]. The by-product of this superimposition is a new set of Procrustes coordinates (or shape coordinates) used for statistical analyses. The GPS was performed with a TPSrelw v. 1.54 [57]. We provide the full morphometric dataset as the electronic supplementary material, including specimen ID, grouping factors and Procrustes coordinates (electronic supplementary material, S2).

### 2.3. Statistical analyses

#### 2.3.1. Anatomical and taxonomic comparisons

To assess whether the shape difference among the four mandibular teeth and all equine taxa were significant, we initially used a factorial MANOVA to account for possible interactions between both anatomical and taxonomic factors. Detecting significant interaction, between the anatomical position of the molar in the mandibular sequence and the taxonomic identification of the specimens, would mean that the taxonomic differences varied according to the anatomical position of the tooth; therefore, discriminant analysis within taxonomic groups had to be performed separately for each tooth.

To assess the assignment accuracy among the four mandibular teeth and the 15 taxa, considered using the shape variation of the enamel outline, we performed a canonical variate analysis (CVA) with a twofold cross-validated classification. This approach calculates the most discriminant axes of variation among the taxonomic grouping factors, following normalization of the intra-group variance by creating a discriminant shape space. Note that the latter is different from the original shape space obtained from a principal component analysis (PCA) which calculates the main axis of variation in the shape coordinates without normalization.

To estimate the performance of the anatomical and taxonomic discriminant models in practice, and their ability to identify the right anatomical position or the right taxonomic group of an isolated tooth, we calculated the percentage of correct classification of each specimen into its known group, be it anatomical or taxonomic, following a twofold cross-validation approach. This consisted of partitioning the dataset into two subsets: the training set on which the analysis was performed, and the testing set that was used to validate the analysis. This procedure is done over multiple rounds (10 000) of cross-validation, changing the dataset partitioning at every round. This provided an average of correct classification for each specimen and a percentage of correct classification, or correct cross-validation (CCV).

To reduce the dimensionality of our shape dataset, we performed a PCA on the shape coordinates before each CVA analysis, and retained the PC scores that explained 99% of the total shape variance.

To visualize shape changes along the main axis of variation (PCA) or along the discriminant axes (CVA), we used a multivariate regression approach [58].

All these analyses were performed with R [54], using the libraries ade4 [59] and geomorph [60]. Graphical displays were performed with MorphoJ [61].

#### 2.3.2. Phylogenetic signal in enamel outline shape

**Figure 3.**
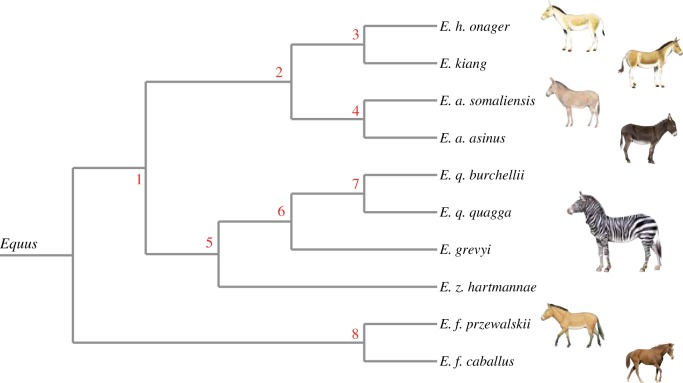
Lineage divergence in equids including the numbering of the internal nodes based on the exome-wide phylogeny [5].

We used the published molecular phylogeny of equids (figure 3; electronic supplementary material, S3) built for 10 taxa: two horses (*E. ferus przewalskii*, *E. f. caballus*), four zebras (*E. quagga quagga*, *E. q. burchellii*, *E. grevyi*, *E. zebra hartmannae*), two asses (*E. africanus somaliensis*, *E. a. asinus*) and two hemiones (*E. h. onager*, *E. kiang*). This phylogeny was obtained applying a maximum-likelihood method on a super-matrix of 20 374 protein-coding genes, each partitioned in 1st + 2nd and 3rd codon position (representing a total of 31 219 584 sites). Node robustness was assessed using 100 bootstrap pseudo-replicates. The analysis was performed using the phylogenetic module of the automated PALEOMIX pipeline [62].

To assess the association between the molecular phylogeny and the dental shape space, we created a phylomorphospace which fitted the shape changes of each tooth from the 10 species onto the tree topology of the molecular phylogeny (including branch length) [49,50]. A dental shape space was constructed for each mandibular tooth of the 10 species using a GPS, followed by the computation of the species mean shape. A PCA was subsequently performed on the matrix of variance covariance of the averaged Procrustes coordinates. To fit the dental shape space onto the phylogeny, we used the squared-change parsimony criterion [63], which finds the PC scores of the shape of the ancestral (root) and internal nodes, for which the changes along the branches of the entire phylogeny (in Procrustes distances units) are minimal [49]. After which, the branches of phylogeny—connecting the ancestral, internal and terminal nodes—can be drawn to estimate the path of the evolutionary lineages in the shape space. The squared-change parsimony approach, which produces the same ancestral shape estimates as maximum-likelihood phylogenetic reconstruction and phylogenetic generalized linear models (PGLM) [64], allows the reconstruction of the ancestral shape at the root of the phylogeny from the shape average of the terminal nodes. It is then possible to graphically visualize the evolutionary changes of the teeth shape from the common ancestor of the *Equus* species, and each internal node and terminal tip of the phylogeny.

To assess the phylogenetic signal in the shape space of the four teeth, we used the *Kmult* method [51]; which is more suited to high-dimensional multivariate data and less affected by the difference of traits variation among species [65]. This method is a multivariate generalization of the *K* statistic which estimates the strength of the phylogenetic signal in a univariate trait relative to a Brownian motion model of evolution [65]. Under a Brownian motion model of evolution the expected *Kmult* value is 1.0. Observed *Kmult* values are evaluated by comparison with the permutated *K* value from randomized shape data relative to the tree topology of the molecular phylogeny.

## 3. Results

The factorial MANOVA found significant differences in shape among the four cheek teeth and the 15 equine taxa investigated (table 2). Although the taxonomic signal was stronger than the anatomical signal, with 21.8% against 13.5% of shape variation explained, respectively, significant interaction between both factors was found to explain 14% of the overall variation. This indicates that the taxonomic differences vary across the four cheek teeth and therefore a separate investigation of the taxonomic resolution for each tooth is required.

**Table 2.** Results of the factorial MANOVA which tested the differences between the four mandibular cheek teeth (anatomical position) and the 15 taxa of our dataset (table 1), as well as the interaction between the two factors (anatomical position : taxa).

	d.f.	SS	MS	Rsq	*F*	*Z*	Pr (>F)
anatomical position	3	1.3784	0.45948	0.13564	41.2	18.452	0.001**
taxa	14	2.2202	0.15859	0.21848	14.2198	7.7823	0.001**
anatomical position : taxa	42	1.4222	0.03386	0.13995	3.0362	1.7319	0.001**

### 3.1. Anatomical distinction of equine mandibular cheek teeth excluding P/2 and M/3

**Figure 4.**
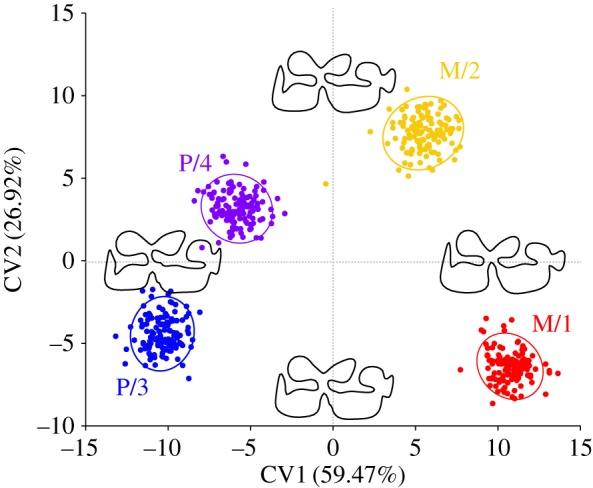
Canonical variate analyses (CVA) comparing shapes P/3, P/4, M/1 and M/2 in equids with visualization of shape differences along the canonical axes and shape reconstruction at the extreme values of each axis.

The discriminant model (figure 4) found correct anatomical identification for each mandibular tooth across the 15 taxa dataset for 93% of the classifications after cross-validation. This shows that this model can accurately identify the anatomical position of any isolated mandibular tooth of the genus *Equus* from the archaeological record with a false-positive probability of only 7%. Misclassifications were systematically between M/1 and M/2 (37%) and between P/3 and P/4 (49%). The remaining 14% were random.

The visualization of the shape changes along canonical axes 1 and 2 (figure 4) showed that the main differences between premolars and molars were a more penetrating ectoflexid, and consequently, a less wide postflexid in the latter. In addition, the premolars displayed flatter proto- and hypoconids as well as a rounder hypoconulid than the molars.

### 3.2. Taxonomic distinction among equids

The discriminant model of the CVA shows that all four mandibular teeth could distinguish the 15 equine taxa of our dataset (table 1), with more than 95% of correct classification with cross-validation (table 3). Premolars performed better than molars, with P/3 being the most efficient with a 100% cross-validated classification (CCV).

**Table 3.** Results using MANOVA of the differences between the 15 taxa for each mandibular cheek tooth and the percentage of correct cross-validated classification (CCV) for each mandibular tooth.

	Pillai	Approx F	num d.f.	den d.f.	Pr	CCV
P/3	12.439	8.09	896	910	<0.0001	100
P/4	12.581	9.14	896	924	<0.0001	98.47
M/1	12.186	8.08	826	994	<0.0001	95.41
M/2	12.188	7.38	854	938	<0.0001	96.12

**Figure 5.**
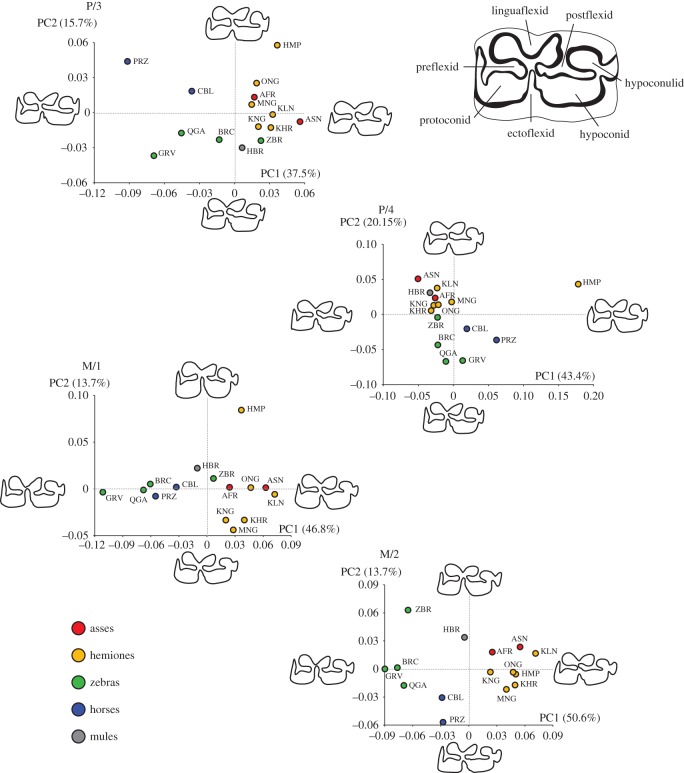
Morphospace for the four mandibular equine cheek teeth. The shape changes of the enamel folding along the major axes of variations (PC1 and PC2) are displayed for the extreme values of each axis. The colour of the dots corresponds to the molecular clades (blue: caballine horses, red: asses, orange: hemiones, green: zebras) and hybrids (grey filling). The three letter abbreviations correspond to the taxonomic groups described in table 1.

The pooled within-group shape space of the 15 taxa studied (figure 5) shows a clear taxonomic signal separating the three clades of zebras, asses and horses. However, the hemippe, also known as the Syrian wild ass (*E. hemionus hemippus*), is divergent from other hemiones in all teeth but the M/2. Mules (donkey × horse hybrids) have a divergent phenotype from horses and asses, except for the P/4 where mules are within the asinine dental variation. The divergence of mules is almost intermediate between horses and wild asses in molars, which strongly suggests that mules could be discriminated in the archaeological record with a statistical predictive approach, should greater sample sizes of mules be available. Such methodological approach could greatly contribute to document the role played by equine hybrids in human history, especially during the Roman and medieval periods [66].

The shape deformation along the main axis of shape variation illustrates how different the morphological criteria are along the location sequence in the tooth row, as previously evidenced by the factorial MANOVA.

(1) For the P/3, the width and shape of the postflexid separates zebras and horses from hemiones and the African wild ass and donkey; while the smoother V-shape of the linguaflexid (double knot) separates horses from zebras, except for *E. z. hartmannae*, which shows a more asinine shape.
(2) For the P/4, the penetration of the ectoflexid is deeper in zebras than in wild and domestic asses and, to a lesser extent, in horses; except for the *E. z. hartmannae*, which again shows a more asinine shape.
(3) For the M/1, the penetration of the ectoflexid up to the linguaflexid fold and flatter protoconid and hypoconid separates zebras and horses from asses; except for the *E. z. hartmannae*, which again shows a more asinine shape. Along the second PC, the kiang (*E. kiang*), the khur (*E. hemionus khur*) and the Mongolian hemione (*E. h. hemionus*) differ from asinines by slender protoconids and hypoconids.
(4) For the M/2, clearer criteria emerge due to a greater structuration of the shape space among the taxa, due to the lack of divergence of the hemippe. Similar to the M/1, the combination of the penetration of the ectoflexid up to the linguaflexid fold and flatter protoconid and hypoconid separates zebras from asses. The latter also shows a wider postflexid up to the preflexid, clearly separating it from zebras and horses. Confirming previous observations from Véra Eisenmann [15], the shape of the linguaflexid can provide criteria to distinguish zebras, horses, asses and hemiones. Asses and hemiones display a V-shaped linguaflexid compared to the more U-shape of horse. Zebras are more intermediate with a smoother V-shape. Horses can be distinguished from zebras due to their less penetrating ectoflexid and their wider postflexid.

### 3.3. Phylogenetic signal in equine mandibular cheek teeth (P/3 to M/2)

**Figure 6.**
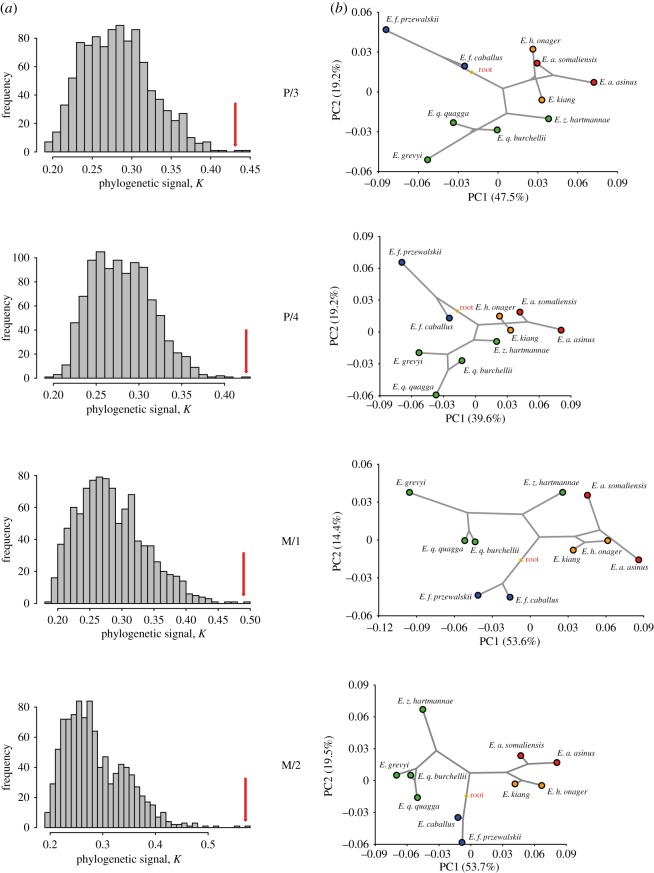
(*a*) Histograms of the *Kmult* values obtained from 1000 permutations of cheek tooth shapes from the tips of the molecular phylogeny with the position of the observed value identified by a red arrow. (*b*) Plot of the phylomorphospace viewed from the first two PCs for the mean shapes of 10 equine taxa onto the tree topology of the molecular phylogeny with a squared-change parsimony approach.

The *K* statistic found significant phylogenetic signals in all four mandibular teeth (table 4 and figure 6*a*). The M/2 has the highest *Kmult* close to 0.6, which suggests that the M/2 shape variation provides the greatest degree of phylogenetic structure among the 10 *Equus* taxa studied. However, the level of phylogenetic signal is still below the *K* value of 1, which suggests that the M/2 shape evolution does not fit an unconstrained Brownian model of evolutionary process.

**Table 4.** Phylogenetic signal from the four mandibular cheek teeth of equids using the *Kmult* method [55].

	*Kmult* obs	Permut *p*-value
P/3	0.432	0.002
P/4	0.4253	0.001
M/1	0.4905	0.001
M/2	0.5711	0.001

Nevertheless, the trend of phylogenetic signals from *Equus* mandibular teeth is supported by the phylomorphospace (figure 6*b*); with related species showing phenotypic similarity to their dental mean shapes, while more distant species are more morphologically divergent. In addition, the dental shape variation seems to diversify from the hypothesized ancestral shape at the root; with extant species occupying a distant region of the phylomorphospace compared with the central ancestral shape, especially for the M/2. Among the four cheek teeth, the M/2 clearly provides the tightest association with the main phylogenetic clades closely related in the shape space with the least discrepency from the phylogenetic tree (figure 6*b*). Finally, the lack of overlapping branches in the phylomorphospace suggests that convergent evolution did not occur during the evolution of the mandibular teeth. However, aside from the M/2, all the cheek teeth of the *E. z. hartmannae* cluster towards the asinine group; though some signs of convergence among asses and hemiones are observable in the P/3 and M/1.

**Figure 7.**
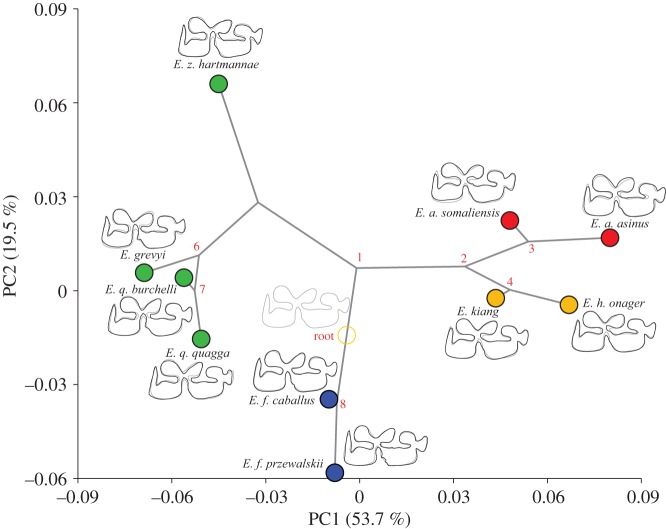
Reconstruction of the evolutionary changes in the shape of M/2 equine enamel folding patterns. The phylogenetic tree from the exome-wide phylogeny has been projected onto the shape space made up of the first two principal components computed from the matrix of variance covariance among the 10 species means. The tips of the branches are the position of the species means in the shape space. The diagrams at the tip of the branches display the changes in the shape of a species (black outline) respective to the reconstructed common ancestor at the root (grey outline), with a scale factor of 1.0.

The first two PCs on the M/2 shape account for almost 75% of the total variation for the 10 species. The reconstruction of the evolutionary changes of the M/2 shape on the exome phylogeny (figure 7) shows a clear divergence between the three clades. The main divergence is in the direction of the PC1 that separates zebras and asinines through the penetration of the ectoflexid of zebras, and the V-shaped linguaflexid and the shoe-shaped entoflexid in asinines. The second divergence observed along the PC2 is the separation of the horse and the diversification among zebras and asses; with the divergence of *E. z. hartmannae* from its sister species and the separation of the African and Asian asinine clades, respectively. The diversification within asinines is marked by a more pronounced V-shaped linguaflexid in asses. Horses are close to the reconstructed common ancestral shape at the root of the tree; their divergence is marked mainly by the greater U-shape of the linguaflexid.

## 4. Discussion

Traditional morphological analyses have so far provided invaluable information to palaeontologists and zooarchaeologists for documenting the evolutionary history of equids, and remain key for future investigation. However, these approaches face two main limitations in their ability to capture the entire geometry of equine teeth and to disentangle the size from the shape component of their form variation. As the environmental impacts on the size variation of the skeleton in mammals [67], it can also potentially confound the phylogenetic and population signals retrieved from morphological data [48,68]. GMM approaches have contributed to resolve these long-lasting caveats by enabling the separate extraction of the size and shape components of biological forms such as teeth, redeeming the use of morphological variation in synergy with palaeogenetic analyses to investigate macro- and microevolution processes in the palaeontological and archaeological records [32,34,46].

Our previous GMM approach of horse upper cheek tooth morphology had already shown that the shape of the occlusal enamel pattern was a reliable character to discriminate populations [45]. It also clearly evidenced the absence of an age-related effect in the shape variation of the occlusal enamel pattern, except for senile and juvenile specimens [45], contradicting previous morphometric studies which considered teeth to be too much affected by wear patterns to provide a reliable discriminant marker between species [40,69]. Here, we improved the quantification of the complex enamel folding patterns in equine mandibular cheek teeth in order to fully explore their potential as taxonomic and phylogenetic markers for future palaeontological and archaeological investigations.

Our GMM approach takes into account the heterogeneous nature of the fossil record, which often consists of isolated teeth. Our discriminant model computed over 15 equine taxa and successfully separated the dental sequence of the four mandibular teeth, with a probability above 90%. This means that the anatomical position in the dental sequence of any archaeological isolated mandibular tooth, except the P/2 and M/3 can now be assessed accurately. In practice, this can be done through a CVA with a predictive approach [70], which aims to assign a set of undetermined objects to a series of four predefined groups (P/3, P/4, M/1, M/2). This approach will compute, initially, the multiple discriminant or canonical functions from the samples defined by the four anatomical groups and will, secondly, apply these canonical functions to the undetermined isolated mandibular teeth from the fossil record. The assignment to their most likely anatomical groups can then be assessed based on their Mahalanobis distances and the *ad hoc* probabilities associated with the four anatomical groups' centroid.

All four mandibular cheek teeth investigated in our study have shown great taxonomic accuracy at the species level, especially the P/3. This suggests that there is a strong taxonomic pattern in the shape of the enamel folding of equids. However, we found that anatomical and taxonomic factors interact, which clearly stresses the fact that each location in the dental sequence will give different phenotypic criteria to identify the species (figure 4). For example, the criterion of the penetration of the ectoflexid up to the linguaflexid folds to separate zebras from asses and horses is only applicable to the molars and not the premolars, supporting previous morphoscopic observations [37]. For the P/3 and P/4, the main criteria separating zebras from asses and horses is the shape of the entoflexid, which appears longer and thinner in zebras and horses than in asses. Again, the U-shape of the linguaflexid to separate the *E. ferus caballus* from zebras, asses and hemiones is mainly visible on the M/2; and appears closer to a smooth V-shape than a true U-shape, which is not obvious for the other mandibular teeth. This dental-specific taxonomic signature probably contributes to the difficulty to perform accurate species assignment when dealing with isolated mandibular teeth [25]. This significant result is specifically useful for archaeozoologists, as it can be concluded that the criteria used previously with the shape of the double knot is valid on the M/2 but not for the premolars.

Among the four mandibular cheek teeth, the P/3 provides complete accuracy in the identification of the 15 equine taxa. However, the M/2 morphospace provides the clearest taxonomic clustering of extant equids, probably because most of the shape space of the other teeth is driven by the divergence of the Syrian wild ass (*E. hemionus hemippus*). The divergent position of hybrid (mules) dental phenotype, between horse/zebra and the asinines phenotypes, has been observed in molars but not premolars. This suggests that phenotypic divergence in equid molars is more congruent with molecular divergence than premolars. The M/2 also provides the strongest phylogenetic signal and the best inference of the equine phylogeny, based on exome sequence data [5]. This demonstrates for the first time that the shape of enamel folds in mandibular teeth encapsulates the phylogenetic relationships within the genus *Equus*, and that related species of *Equus* share similar dental phenotype. However, the evolutionary processes underlying this phylogenetic signal are potentially manifold [71]. The diversification of the related *Equus* taxa from a central common ancestor in a different corner of the phylomorphospace of the M/2 (figure 6) suggests that the evolutionary changes of the enamel fold shape from the 4.5 Myr-old most recent common ancestor of modern *Equus* until the present day, can chiefly be explained by a vicariant speciation during the radiation from the Americas into Eurasia and Africa. It also suggests that most of the morpho-functional changes in the tooth morphology may have happened earlier in equine evolution, probably during the Early–Middle Miocene (20–15 Ma) when equids adapted their tooth morphology to grazing in extensive grasslands of open biomes [9]. The power of GMM for taxonomic discrimination of early equids based on cheek teeth remains, however, to be demonstrated. GMM has proved to be suitable to address non-dental evolution of early taxa of the family [72], but when applied to cheek teeth of crown equids such an approach may be less discriminant due to their less divergent morphology.

However, even the highest *K* value found in an M/2 is below the threshold of a Brownian motion model of evolution; which suggests that genetic drift and randomly fluctuating natural selection over time does not fully account for the dental shape diversification in the *Equus* and that evolutionary conservatism [73,74] in dental variation should be rejected. This absence of complete hierarchical phylogenetic relationships in the dental shape variation suggests that other factors, such as morphological convergence and interspecific hybridization, could have occurred [65], as also suggested from patterns of genomic variation [9]. The morphological proximities observed among distant species such as asses and hemiones or between the mountain zebra (*E. z. hartmannea*) and asinines on all but the M/2 cheek teeth could support this possibility, without disentangling convergence from interspecific hybridization as a causal factor. But the latest study on the speciation process in *Equus* is based on an extensive genome-wide dataset and has provided strong evidence for a speciation gene flow in equids [10]. These authors found four, yet undated, events of gene flow among equine lineages following their divergence: between horses and zebra/ass clades, between kiang and donkey, between the African wild ass and Grévy's zebra, and between asses and mountain zebra. This clearly supports gene flow as one of the factors explaining the incomplete hierarchical phylogenetic relationships in *Equus* dental shape variation.

## 5. Conclusion

This comparative study provides clear evidence for the relevance of dental phenotypes to accurately discriminate between all modern members of the genus *Equus* and capture their phylogenetic relationships. Our work redeems the use of dental morphology to explore, in the fossil record, the spatial and temporal dynamics of the equine evolutionary history during the Plio–Pleistocene; and opens up total evidence approaches integrating fossils with molecular phylogeny to improve our understanding of their phenotypic evolution. In the Late Pleistocene and Holocene fossil record of the Old World, the implementation of such a comparative approach will provide an unprecedented opportunity to explore the diversity of the wild ancestors of the domestic horse and donkey, and how these relate to the earliest archaeological evidence of domestic forms.

## Supplementary Material

Specimens details

## Supplementary Material

Geometric morphometric dataset

## Supplementary Material

Genome phylogeny

## Data accessibility

The data supporting this article are available in the electronic supplementary material.
